# Yeast Diversity on Sandy Lake Beaches Used for Recreation in Olsztyn, Poland

**DOI:** 10.3390/pathogens14080744

**Published:** 2025-07-29

**Authors:** Tomasz Bałabański, Anna Biedunkiewicz, Jan P. Jastrzębski

**Affiliations:** 1 Department of Microbiology and Mycology, Faculty of Biology and Biotechnology, University of Warmia and Mazury in Olsztyn, Oczapowski 1A St., 10-719 Olsztyn, Poland; tomasz.balabanski@uwm.edu.pl; 2 The Center for the Popularization of Science and Innovation of the University of Warmia and Mazury in Olsztyn ″Kortosfera″, Dybowski 11A St., 10-723 Olsztyn, Poland; 3 Department of Plant Physiology, Genetics and Biotechnology, Faculty of Biology and Biotechnology, University of Warmia and Mazury in Olsztyn, Oczapowski 1A St., 10-719 Olsztyn, Poland; jan.jastrzebski@uwm.edu.pl

**Keywords:** microfungi, yeasts, sand, beaches, lake ecosystems, sand pollution

## Abstract

Yeasts possess a range of environmental adaptations that allow them to colonize soil and sand. They can circulate seasonally between different components of lake ecosystems, including beach sand, water, and the coastal phyllosphere. The accumulation of people on beaches promotes the development and transmission of yeasts, posing an increasing sanitary and epidemiological risk. The aim of this study was to determine the species and quantitative composition of potentially pathogenic and pathogenic yeasts for humans present in the sand of supervised and unsupervised beaches along the shores of lakes in the city of Olsztyn (northeastern Poland). The study material consisted of sand samples collected during two summer seasons (2019; 2020) from 12 research sites on sandy beaches of four lakes located within the administrative boundaries of Olsztyn. Standard isolation and identification methods used in diagnostic mycological laboratories were applied and are described in detail in the following sections of this study. A total of 259 yeast isolates (264, counting species in two-species isolates separately) belonging to 62 species representing 47 genera were obtained during the study. Among all the isolates, five were identified as mixed (two species from a single colony). Eight isolated species were classified into biosafety level 2 (BSL-2) and risk group 2 (RG-2). The highest average number of viable yeast cells was found in sand samples collected in July 2019 (5.56 × 10^2^ CFU/g), August, and September 2020 (1.03 × 10^3^ CFU/g and 1.94 × 10^3^ CFU/g, respectively). The lowest concentrations were in samples collected in April, September, and October 2019, and October 2020 (1.48 × 10^2^ CFU/g, 1.47 × 10^2^ CFU/g, 1.40 × 10^2^ CFU/g, and 1.40 × 10^2^ CFU/g, respectively). The results indicate sand contamination with yeasts that may pose etiological factors for human mycoses. In light of these findings, continuous sanitary-epidemiological monitoring of beach sand and further studies on its mycological cleanliness are warranted, along with actions leading to appropriate legal regulations.

## 1. Introduction

Yeasts exhibit high physiological and ecological plasticity, and show a wide tolerance to various environmental factors such as temperature, UV radiation, light, humidity, salt content, and conditions of limited availability of nutrients and water [1,2,3,4,5,6,7,8]. These properties have allowed microfungi to achieve ecological success and colonize most ecosystems of the biosphere, including water and soil, which is also reflected in the 2021 WHO guidelines that recognize beach sand as a relevant ecological reservoir of fungi in recreational areas [1]. Studies have demonstrated the presence of yeasts in a variety of environments, including marine, deep-sea, and freshwater reservoirs, aquatic ecotone habitats, ontocenoses of aquatic organisms, the surfaces of coastal phyllosphere, and bottom sediment layers of water bodies, as well as sand from beaches and coastal areas [1,9,10,11,12,13,14,15,16,17,18]. The ability of yeasts to thrive in such a broad range of habitats, from extreme marine conditions to terrestrial and freshwater ecosystems, highlights their versatile metabolic pathways and resilience to fluctuating environmental factors [4,5,7,8].

**Figure 1:**
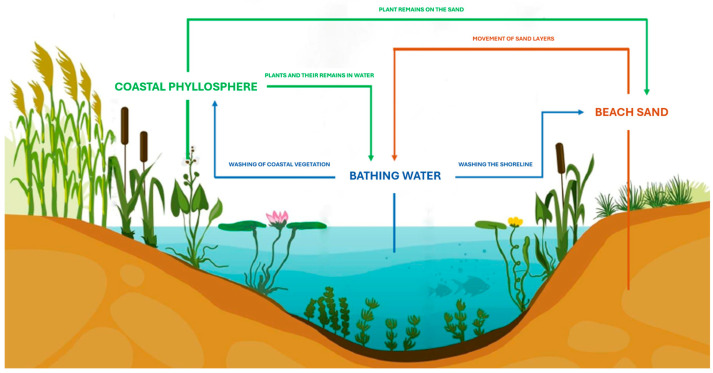
Seasonal circulation of yeasts in lake ecosystem [10].

Yeasts can not only inhabit but also circulate between different components of lake ecosystems, including beach sand, water, and coastal phyllosphere (Figure 1). They migrate from soil or sand into water through surface and subsurface runoff, temporarily settling on coastal phyllosphere, which acts as a natural filter for the water body (Figure 1). Humans, as beach and swimming area users, can serve as temporary vectors for the transmission of microfungi, transferring allochthonous species for which lake ecosystem components are not natural reservoirs [1,7,10,13,19,20,21,22]. This concern has been emphasized in the 2021 WHO guidelines, which recommend monitoring beach sand as a potential source of exposure to opportunistic fungi [1].

Soil and beach sand, in particular, represent environments of interest. Yeasts are a significant component of the taxonomic structure of microbiological soil biomass. Their adaptive capabilities enable them to thrive in soil ecosystems, which includes sand, where yeasts can adhere to granules in suspension or as biofilms [11,20,23,24].

The yeast nutrition method based on primary osmotrophy is a crucial factor in shaping the above-mentioned adaptive abilities. They secrete adaptive exoenzymes into the environment to break down nutritional substrates, with the resulting products then being absorbed into the cells via osmosis. When even small amounts of a new substrate appear or the proportions of existing ones change, yeasts stimulate their enzymatic apparatus to produce new exoenzymes. These properties make microfungi, including yeasts, key players in essential ecological processes such as the biogeochemical cycling of elements in nature [5,25,26].

A negative aspect of such advanced adaptive capabilities is the ability to cause diseases in humans. Research shows a continuous increase in the occurrence of yeasts in the human body, including species not previously associated with ontocenoses of the human body [8,27,28,29,30].

Mycological studies of lake ecosystems involve determining the species, taxonomic, and quantitative structure of microfungi and examining their biochemical and ecological activities. These parameters provide an overview of the sanitary and ecological condition of the water reservoir and the processes occurring within it, which play an important role in analyzing the dynamics of these ecosystems. Detailed analysis of these factors allows for the assessment of the sanitary-epidemiological status of the studied environments, related risks, and the development of rational water management plans, which aligns with the recommendations of the WHO, emphasizing the importance of including beach sand and fungal components in routine microbiological assessments of recreational water environments [1,10,31].

The aim of the study was to determine the species and quantitative composition of the overall yeast population present in the sand of supervised and unsupervised beaches along the shores of lakes in the city of Olsztyn (northeastern Poland), including species that are potentially pathogenic and pathogenic to humans. This research focus is relevant from both an ecological and epidemiological perspective, as beach sand may serve as a reservoir and transmission route for opportunistic yeasts, some of which can cause infections in humans, particularly in immunocompromised individuals or those with skin injuries. Moreover, their presence may reflect broader environmental imbalances and pose a public health concern in recreational freshwater areas. The research was based on the hypothesis that among the yeast species occurring in beach sand, there would be strains potentially harmful to human health, which may pose a risk to beach users.

## 2. Materials and Methods

### 2.1. Research Area and Material

**Figure 2:**
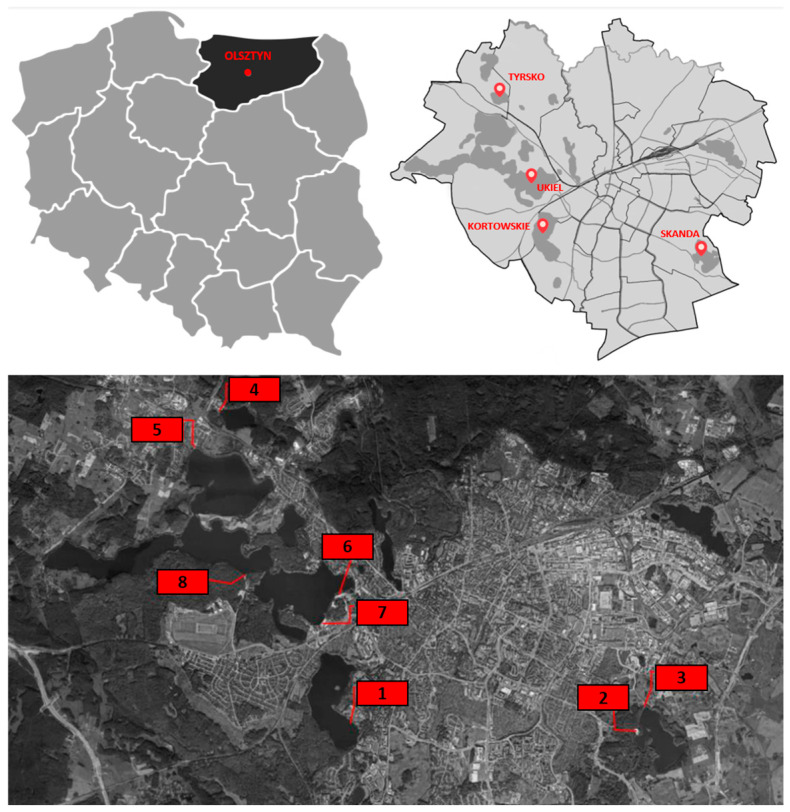
Location of research sites against the background of the administrative map of Olsztyn (northeastern Poland) 1—Beach on Lake Kortowskie; 2—Beach on Lake Skanda; 3—Beach on Lake Skanda; 4—Beach on Lake Tyrsko; 5—Beach on Lake Ukiel; 6—Beach on Lake Ukiel (“Miejska”); 7—Beach on Lake Ukiel (“Omega”); 8—Beach on Lake Ukiel (“Słoneczna Polana”).

The research material consisted of yeasts isolated from sand during two summer seasons (April to October 2019 and 2020) from eight research sites located on supervised and unsupervised sandy beaches along the shores of lakes within the administrative borders of Olsztyn (northern Poland, Europe) (Figure 2). Four lakes were selected for the study: Kortowskie, Skanda, Tyrsko, and Ukiel, whose bathing waters undergo routine bacteriological testing by authorized state institutions (Provincial Sanitary and Epidemiological Station and County Sanitary and Epidemiological Station). Four of the beaches included in the study (sites 3, 6, 7, and 8) are supervised by authorized municipal institutions (Figure 2).

### 2.2. Sampling

Sampling sites were designated at the midpoint of the shoreline of each beach, 3 m from the water’s edge. Fifty-gram sand samples were collected from two depths (10 cm and 50 cm) using a soil auger (Fiskars) sterilized with a 70% alcohol solution. For sample unification, a 1 m × 1 m square was marked at the designated location, and 5 soil cores were taken (one from each corner of the square and one from the intersection of the diagonals). From each core, 10 g of soil were collected into a sterile Ziplock bag, sealed, and mixed to obtain a unified 50 g sample. Samples were collected during the first week of each month of the research season, twice a day (in the morning, 8:00–10:00 a.m., and in the evening, 8:00–10:00 p.m.). During sampling, the presence of beach users varied depending on the time of year. In the spring and summer months, a higher number of people were typically present on the beaches, particularly during afternoon and evening hours, which could potentially influence microbial loads. However, during periods of legal restrictions related to the COVID-19 pandemic—when access to public recreational infrastructure was limited or entirely prohibited—no beach users were present at the study sites due to the enforcement of national sanitary regulations. During two research seasons, a total of 448 sand samples of 50 g were collected from 8 study sites. During sample collection, air temperature and humidity were measured, and the UV index was recorded (Appendix A). Until yeast isolation, the collected sand samples were stored under refrigeration (4 °C) [32].

### 2.3. Yeast Isolation and Cultivation

The yeast isolation procedure was carried out within 48 h of sand sample collection. Yeasts were isolated from the sand samples using a modification of methods described in previous studies [32,33]. From the 50 g sand samples, 10 g sand samples were placed in 90 mL of sterile tap water (1:10 dilution) and shaken for 15 min. Yeasts were isolated by surface inoculation of 0.1 mL of the shaken suspension on Bengal Rose Agar with chloramphenicol (RBC) [32,33,34]. Each inoculation on the RBC medium was performed in triplicate. Given that 448 sand samples were tested, the total number of inoculations was 1344. In pilot studies, the tested media included RBC agar, Sabouraud agar with chloramphenicol, PDA (potato dextrose agar), and Czapek-Dox agar. The highest efficiency in yeast isolation from the studied sites was achieved using RBC medium. This medium was selected for further research due to the selective properties of Rose Bengal. Pilot studies demonstrated that RBC medium effectively inhibits the growth of filamentous fungi, particularly rapidly growing species such as *Rhizopus* and *Mucor*, thereby facilitating the growth and isolation of yeasts. Inoculated media were incubated at 25 °C for 48–72 h. In pilot studies conducted to validate the method, no growth was obtained at 37 °C, so a 25 °C incubation temperature was adopted for effective isolation in further studies. After incubation, macroscopic and microscopic evaluations of the colonies were conducted to differentiate yeast colonies from bacterial and filamentous fungal colonies. The yeast colonies were counted and passaged onto Sabouraud agar slants with chloramphenicol and incubated at 25 °C for 48 h. After incubation the cultures were stored under refrigeration (4 °C) for further studies.

### 2.4. Yeast Count Assessment

To assess yeast population density in the sand, the plate technique using solid media was employed [32]. According to the method, the number of viable cells in the sample is equal to the number of observed colonies. The results were calculated as colony-forming units (CFU) per 1 g of dry soil mass.

### 2.5. Yeast Identification

A macromorphological description of the verified yeast colonies (i.e., colonies that were microscopically confirmed as yeasts) was made, including features such as shape, color, consistency, surface prominence, edge structure, transparency, smell, and growth into the medium. The ability of the isolated yeasts to ferment a basic carbohydrate series (glucose, galactose, maltose, sucrose, lactose) was tested using a liquid medium for carbohydrate zymogram (tested sugar 20 g; peptone 0.5 g; distilled water 1000 mL; bromothymol blue with NaOH to achieve a blue color). Suspensions of the colonies of the tested strains were prepared in sterile tap water with turbidity of 3° on the McFarland scale. A 40 µL drop of the suspension was added to the zymogram medium tubes and incubated at 25 °C for 48 h. Fermentation ability was indicated by a change in the color of the solution from blue to yellow (lowering of the medium’s pH due to fermentation) and/or medium turbidity (increased yeast biomass).

To analyze micromorphological features, microcultures were prepared on Nickerson’s agar [8,31,35,36]. Microcultures were set up in sterile moist chambers by passaging colony inoculum onto the surface of Nickerson’s agar, followed by covering it with 20 µL of rabbit serum solution with bacteriological broth (1:1). The inoculum was then covered with a sterile coverslip. To maintain appropriate humidity, 1 mL of sterile distilled water was placed at the bottom of the chamber. The microcultures were incubated at 25 °C for 144 h to reveal diagnostically significant features, such as the size, shape, location, and formation of blastospores, the ability to produce chlamydospores, pseudohyphae, and true hyphae, as well as their size, shape, and arrangement [8,10,13,35,36]. Microculture on Nickerson’s agar remains a relatively underutilized yet highly valuable method for yeast morphology assessment and identification. This technique enables precise species determination by leveraging knowledge of the specific and spatial growth patterns of mycelium or pseudomycelium, allowing for natural, undisturbed development without external interference. Its ability to preserve the inherent structural characteristics of fungal growth makes it a crucial tool for accurate taxonomic differentiation [8,10,13,35]. Microscopic observations of the microcultures were made at 48, 72, and 144 h of incubation, with photographic documentation at each stage.

For strains requiring additional data for identification, Schaeffer–Fulton staining was performed to demonstrate the presence of asci with ascospores and to classify the strain as either ascomycetous or non-ascospore-forming yeasts [37].

The classification of strains into biosafety levels (BSL) and risk groups (RG) was based on multiple sources, as data on these categories vary across references and countries. However, diagnostic keys served as the primary basis for classification, with additional literature consulted to ensure accuracy and reliability [19,38,39,40].

To achieve more precise identification for a subset of the studied strains, molecular analyses were conducted in addition to phenotypic methods (including macro- and microculture observations). Genomic DNA was isolated using the Genomic Mini AX Yeast Spin kit (A&A Biotechnology, Gdańsk, Poland). Based on the convergence of phenotypic traits and selected biochemical analyses, 49 of the most representative samples (the most frequently occurring species identified phenotypically) were selected for molecular analysis to avoid redundant testing of multiple strains of the same species. PCR (polymerase chain reaction) was performed, followed by purification and sequencing of the ITS-1 (5′-TCCGTAGGTGAACCTGCGG-3′) and ITS-4 (5′-TCCTCCGCTTATTGATATGC-3′) regions of DNA for selected yeast isolates. The resulting nucleotide sequences were compared with the GenBank database using the Nucleotide BLAST tool (BLASTN, NCBI online version accessed in January 2025; https://blast.ncbi.nlm.nih.gov). The sequences used for identification have been deposited in the GenBank database under accession numbers PQ882533–PQ882625.

Final species classification of strains was conducted by integrating results from both phenotypic and molecular methods. In cases of uncertainty, final identification was achieved by comparing microculture observations on Nickerson’s agar with diagnostic keys [19,38,39].

### 2.6. Statistical Analysis

Statistical analysis was performed using STATISTICA 13.3 software (StatSoft). The following tests were used in the analysis: nonparametric Mann–Whitney U test, nonparametric Kruskal–Wallis H test, one-way ANOVA (analysis of variance), Tukey’s b test, paired Student’s *t*-test (for comparing the number of isolates per species between the 2019 and 2020 seasons), and Spearman’s rank correlation coefficient. For all tests, the significance level was set at *p* < 0.05.

## 3. Results

During the study, a total of 259 yeast isolates were obtained, representing 62 species from 47 genera (Table 1). Thirty-two of the identified genera belonged to ascomycetes (68.1%), and the remaining fifteen to basidiomycetes (31.9%) (Table 1). Among all the isolates, five were two-species isolates (19.3% of all isolates) (Table 1 and Table 2). Species from two-species isolates were counted separately in both quantitative and statistical analyses (Table 1 and Table 2). Of the sixty-two species, eight were classified as BSL-2 and RG-2 (12.9%) (Table 1). Preliminary macroscopic selection, including the macromorphological features of the colonies, indicated the need to distinguish different isolates, which were later classified as the same species during further diagnostic stages.

Analysis of individual research seasons showed differences in the number of isolates obtained from the beaches. Overall, more isolates were obtained in the second research season (2020; 149 isolates, including two two-species isolates), while 86 fewer isolates were obtained in the first research season (2019; 63 isolates, including three two-species isolates) (Table 1 and Table 2). The difference in the number of isolates was not statistically significant (*p* = 0.0927). Similarly, the paired *t*-test (*p* = 0.170) revealed no statistically significant difference in the average number of isolates per sampling event between the two seasons, although a tendency toward higher isolation frequency was observed in 2020 (mean = 4.45) compared to 2019 (mean = 3.43). This may reflect differences in environmental conditions or anthropogenic pressure.

In the total number of isolates, those obtained from sand in the morning hours predominated (147 isolates, 55.7% of all isolates). Fewer yeasts were isolated from sand collected in the evening (117 isolates, 44.3% of all isolates) (Table 1). No statistically significant differences were found in the number of isolates obtained in the morning and evening (*p* = 0.7955).

Larger differences were observed in the case of sand samples collected from different depths (Table 1). In the total pool of isolates, yeasts collected from a depth of 10 cm clearly dominated (175 isolates, 66.3% of all isolates). Fewer yeasts were isolated from soil at a depth of 50 cm, below the mixing layer of the sand (89 isolates, 33.7% of all isolates) (Table 1). These differences, however, were not statistically significant (*p* = 0.0927).

From the beaches supervised by authorized municipal institutions, 100 isolates were obtained (37.9% of all isolates), while 164 isolates were obtained from unsupervised beaches (62.1% of all isolates) (Table 1). These differences were not statistically significant (*p* = 0.2131).

**Figure 3:**
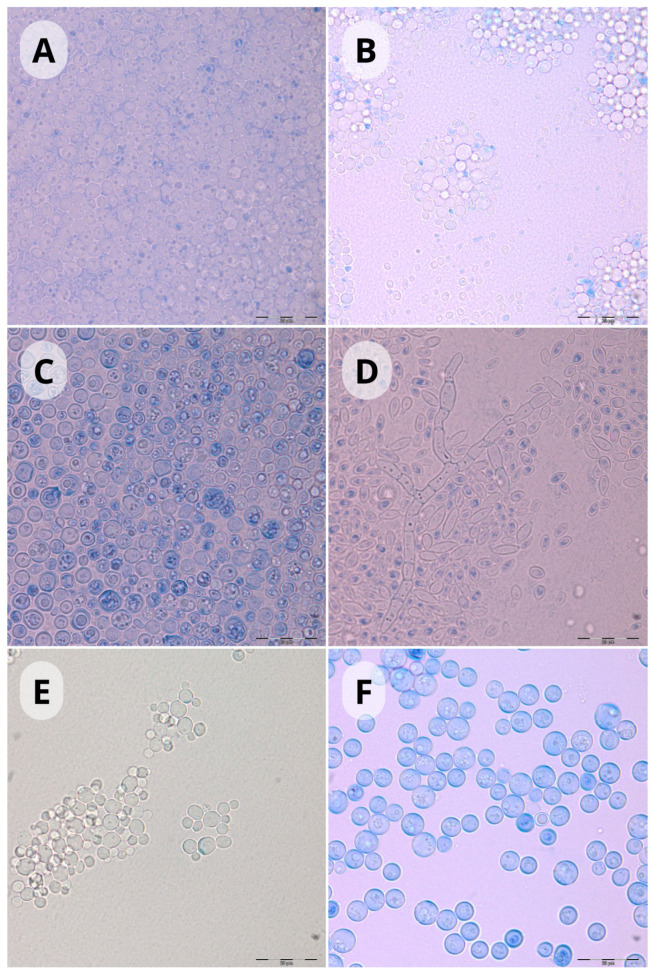
Most often isolated species (microcultures on Nickerson’s agar in moist chambers, incubated at 25 °C for 144 h) (**A**)—*Barnettozyma californica* 1000×; (**B**)—*Nakasemomyces glabratatus* 1000×; (**C**)—*Cutaneotrichosporon jrovecii* 1000×; (**D**)—*Cutaneotrichosporon moniliiforme* 1000×; (**E**)—*Debaryomyces hansenii* 1000×; (**F**)—*Solicoccozyma aeria* 1000×. Scale bar: 20 µm.

Among the yeasts isolated during the study, the most frequently represented species were: *Barnettozyma californica*, *Nakasemomyces glabratatus*, *Cutaneotrichosporon moniliiforme*, *C. jirovecii*, *Debaryomyces hansenii*, and *Solicoccozyma aeria*. Other species occurred in smaller numbers (eight isolates or less) (Table 1, Figure 3).

In the 2019 season, the most frequently isolated species were: *B. californica*, *Cutaneotrochosporon jirovecii*, *C. moniliiforme*, *Schwanniomyces capriottii*, and *Tausonia pullulans* (Table 1) Among the species isolated in the 2020 season, *D. hansenii*, *Mycogloea nipponica*, *N. glabratatus,* and *S. aeria* dominated (Table 1). Twenty-one species and five strains identified only to genus (one genus) were isolated in both research seasons, representing 33.3% of the species and genera pool obtained during the study. Twelve species and one strain identified only to genus one1 genus) were noted exclusively in the 2019 season (19.7% of the total species and genera pool). Twenty-nine species and four strains identified only to genus (two genera) were found only in the 2020 season (46.9% of the total species and genera pool) (Table 1).

From morning-collected sand samples, the most frequently isolated species were: *B. californica*, *Cutaneotrichosporon jirovecii*, *C. moniliiforme*, *D. hansenii*, and *S. aeria* (Table 1). In the evening-collected sand, the most common species were: *C. glabrata*, *Cutaneotrichosporon jirovecii*, *C. moniliiforme*, and *D. hansenii* (Table 1). Thirty-two species and nine strains identified to genus (two genera) were isolated both in the morning and evening, representing 51.5% of the total species and genera pool detected during the study (Table 1). Seventeen species and one strain identified to genus (one genus) were found only in the morning samples (27.3% of the total species and genera pool). Thirteen species and one genus were found only in evening samples (21.2% of the total species and genera pool) (Table 1).

From a depth of 10 cm, the most frequently isolated species were: *B. californica*, *Citeromyces matritensis*, *Cutaneotrichosporon jirovecii*, *C. moniliiforme*, *D. hansenii*, *N. glabratus*, and *S. aeria* (Table 1). Among species isolated from a depth of 50 cm, *N. glabratus*, *C. moniliiforme*, and *D. hansenii* dominated (Table 1). Twenty-four species and seven strains identified only to genus (two genera) were isolated from both depths (10 cm and 50 cm), representing 39.4% of the total species and genera pool (Table 1). Twenty-eight species and one genus were found only in the 10 cm deep samples (43.9% of the total species and genera pool). Ten species and one genus were isolated only from the 50 cm deep samples (16.6% of the total species and genera pool) (Table 1).

In the sand from supervised beaches, the most frequently isolated species were: *B. californica*, *N. glabratus*, *C. moniliiforme*, and *D. hansenii* (Table 1). From the sand of unsupervised beaches, the most common species were: *Cutaneotrichosporon jirovecii*, *C. moniliiforme*, *D. hansenii*, and *S. aeria* (Table 1). Twenty-six species and one genus were isolated from both types of beaches (supervised and unsupervised), representing 40.9% of the total species and genera pool detected during the study (Table 1). Twelve species and two genera were noted only in samples collected from supervised public beaches (21.2% of the total species and genera pool). Twenty-four species and one genus (strain identified only to genus) were found only in the sand from unsupervised beaches (37.8% of the total species and genera pool).

During the two-year study, the most isolates were obtained from the beaches of Lake Skanda (87 isolates; 32.95% of all isolates) and Lake Ukiel (87 isolates; 32.95% of all isolates). From Lake Kortowskie, 63 isolates were recorded (23.9% of all isolates). The fewest isolates were from Lake Tyrsko (27 isolates; 10.2% of all isolates) (Table 1).

Taking into account the strains identified to the species level, the greatest taxonomic diversity was observed in the case of Lakes Ukiel and Skanda (33 and 32 isolated species, respectively), and the least in the case of Lake Tyrsko (19 isolated species). From the sand of the beaches of Lake Kortowskie, 27 species were isolated (Table 1). The obtained results allowed for a detailed characterization of the species structure of the sand on the beaches of individual lakes (Table 1). Nine species were found exclusively in the sand of the beaches of Lake Kortowskie, the most frequently isolated was *Papiliotrema pseudoalba*. Seven species were isolated exclusively from the sand of the beaches of Lake Skanda, with *Exophiala jeanselmei*, classified as BSL-2 and RG-2, being the most frequent. Four species were found only in the sand of the beach of Lake Tyrsko, with *Exophiala castellanii*, also classified as BSL-2 and RG-2, being the most frequent. In the case of Lake Ukiel, nine species and five strains identified only to the genus (three genera) were noted exclusively in the sand of the beaches of this lake, with *Nadsonia commutata* being the dominant species.

Three species were recorded in the sand of the beaches of all the lakes (*Cutaneotrichosporon jirovecii*, *C. moniliiforme*, and *D. hansenii*). Two of them, *C. jirovecii* and *C. moniliiforme*, were classified as BSL-2 and RG-2 (Table 1). Fourteen species were found in the sand of the beaches of three out of the four studied lakes, including one (*N. glabratus*, formerly *C. glabrata*) classified as BSL-2 and RG-2 (Table 1). Fourteen species were isolated from the sand of the beaches of two lakes, while 35 species were found in the sand of beaches located near only one lake (Table 1). Five of the species present in the sand of the beaches of only one lake (*Candida albicans*, *Clavispora lusitaniae*, *Exophiala bergeri*, *E. castellanii*, and *E. jeanselmei*) were classified as BSL-2 and RG-2 (Table 1).

**Figure 4:**
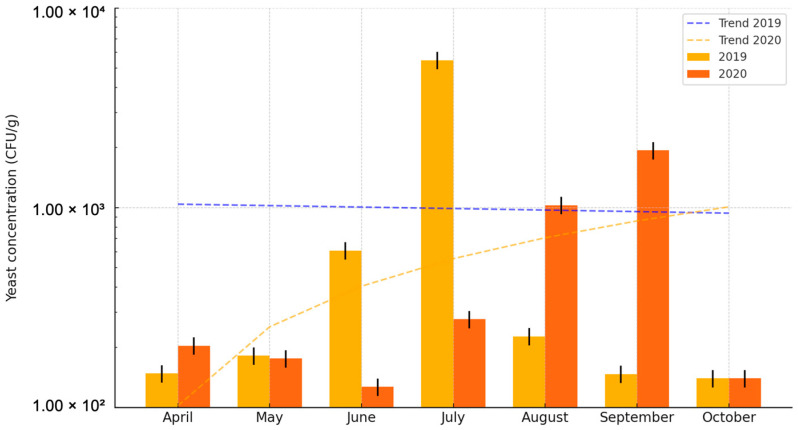
Average yeast concentration (CFU/g) in sand from monitored beaches across both research seasons (2019–2020), presented on a logarithmic scale. Error bars represent estimated variability (±10% of the measured values).

The number of viable cells of individual strains was counted, and the obtained data were compiled in tabular form (Table S1). On average, nearly twice as many viable yeast cells were isolated in the 2019 season (9.91 × 10^2^ CFU/g) compared to the 2020 season (5.56 × 10^2^ CFU/g) (Table S1; Figure 4). The fluctuations in the average number of yeasts in 1 g of sand in the 2019 season ranged from 1.40 × 10^2^ CFU/g in October to 5.48 × 10^3^ CFU/g in July, while in the 2020 season, they ranged from 1.40 × 10^2^ CFU/g in October to 1.94 × 10^3^ CFU/g in September. The highest average number of viable yeast cells was found in sand samples collected in July 2019 (5.56 × 10^2^ CFU/g), and in August and September 2020 (1.03 × 10^3^ CFU/g and 1.94 × 10^3^ CFU/g, respectively). The lowest concentrations were in samples collected in April, September, and October 2019, and in October 2020 (1.48 × 10^2^ CFU/g, 1.47 × 10^2^ CFU/g, 1.40 × 10^2^ CFU/g, and 1.40 × 10^2^ CFU/g, respectively) (Table S1; Figure 4). In 2019, compared to 2020, more viable yeast cells were isolated in May, June, and July. In July 2019, the average number of viable yeast cells was an order of magnitude higher than in the 2020 season. The opposite trend was observed in April, August, and September. In August and September 2020, the average number of viable yeast cells was an order of magnitude higher than in the corresponding months of 2019. In October of both seasons, the same average number of viable yeast cells was recorded (Table S1; Figure 4). The differences in the average concentration of strains between individual months were statistically significant (*p* = 0.0300). Post hoc analysis showed that this difference occurred between April and July (the test considered numerical data from both research seasons and all studied beaches).

Statistical analysis was conducted to assess the impact of air temperature, air humidity, and UV index on the number of isolates and strain concentrations in the sand during individual months of the research seasons, revealing that only air temperature significantly affected the average strain concentration in the sand (*p* = 0.0210) (Table 3). The potential impact of air temperature on the abundance of the yeast population in the soil is justified in the later sections of this study.

## 4. Discussion

Research shows high ecological and taxonomic diversity of yeasts found in soil ecosystems [7,20,41]. The taxonomic structure of yeasts in sand is very uneven and diverse. It includes many species, classified both in the phylum *Ascomycota* and *Basidiomycota*. There is a relationship between the taxonomic and numerical structure of soil yeast populations and the cross-section of the soil profile [7,41]. The results of this study confirm this observation. According to the results, the largest number of yeasts occurs at a depth of about 10 cm, although their number is quite variable and may range from 10 to over 100 CFU (colony-forming units) per gram of dry soil mass. A clear correlation was found between the size of yeast populations and the content of carbon and nitrogen in the soil [7,20]. Thanks to their adaptive abilities, soil and sand provide good environments for yeast development due to the favorable microclimate, richness in available carbon and nitrogen sources, as well as other macro- and microelements [7,11,19,42].

Sanitary-epidemiological monitoring of recreational beaches currently includes only various parameters of bathing waters. This legal issue has been pointed out in other studies [42,43,44]. Other authors emphasize the unique and complex structure of sand, which requires going beyond standard beach monitoring parameters and developing independent quality control standards and microbiological norms to ensure the safety of beach users [45]. This view is supported by the 2021 WHO guidelines on recreational water quality, which recognize beach sand as a potential source of microbiological risk—including fungi—and recommend its inclusion in routine environmental monitoring of recreational sites [1,46].

Quantitative analysis and species pool of microfungi in beach sand depends on numerous atmospheric and environmental factors [11,42,43,45]. The present study found a statistically significant effect of air temperature on the average concentration of strains in the sand (higher temperature resulted in a higher average strain concentration). Other studies confirm the influence of air temperature on the temperature within the soil profile in its shallower layers. This influence decreases with depth [46]. This suggests that air temperature may have at least an indirect impact on the numerical and taxonomic structure of the yeast population in the upper layers of the soil profile. Other authors highlight the direct impact of temperature on the survival of microorganisms in water and sand [45,47]. Moreover, they suggest a possible connection between global warming and the growth kinetics of microorganisms, pointing out that, despite the general belief that increased temperatures favor microbial growth, in the case of mesophilic microorganisms, it will act as a limiting factor, while favoring the growth of pathogens that prefer conditions closer to the human body temperature [45]. An example could be yeasts of the genus *Candida*, which exhibit xerotolerant properties [48]. Furthermore, it is noted that increased temperatures affect other environmental parameters that shape the microbiological structure of beaches [45]. For instance, an indirect negative correlation between the abundance of fungi in sand and the length of exposure to sunlight has been demonstrated, with the latter causing an increase in sand temperature [11]. Similar to the present study, no statistically significant effect of air humidity on the number of yeasts per gram of soil was observed [11].

There were no clear statistical differences in the total number of isolates or overall strain concentration between the 2019 and 2020 seasons. Nevertheless, the data suggest a tendency toward a higher frequency of yeast isolation in 2020 compared to 2019. This trend may reflect seasonal variation in environmental factors such as temperature and humidity, as well as differences in anthropogenic pressure between the two research seasons. A broader analysis across individual months of the research season (April–October) also indicated that while monthly fluctuations in isolate counts were observed, they were not substantial. However, more pronounced variation was noted in strain concentrations between specific months, particularly in early and mid-summer. Similar findings have been reported by other authors, who emphasize that increased human activity during the peak summer months—including recreational use of beaches and the organization of public events—may lead to elevated microbial contamination of beach environments [45]. The positive correlation between the concentration of microfungi in beach sand and the number of users is confirmed by other studies [49,50]. This issue, however, requires further analysis, especially in the context of the number of users, due to incomplete data on population flow provided by supervisors of the studied municipal beaches (Appendix B). Additionally, an analysis of the average concentration of yeasts in the sand of beaches across studied lakes was conducted. In both research seasons, the lowest average concentration of yeasts in the sand was recorded for the lake with the smallest surface area. This observation is likely due to the varying number of beach users, which correlates with the size and availability of recreational areas at the studied water bodies. Lake Tyrsko has the shortest shoreline, significantly limiting the bathing area and the number of users. Other researchers have pointed out the possible influence of the water body’s structure on the taxonomic and numerical composition of yeast populations in the associated ecological niches [10,13,22].

There is no statistical relationship between the season and the number of isolates or the concentration of yeasts in the soil. Similar results were presented in the other study [11]. This suggests that the numerical structure of yeasts in beach sand is determined not by the season, but rather by long-term atmospheric conditions.

Moreover, no significant changes in the concentration and number of yeast isolates were observed between the times of day studied (morning; evening). The present study correlates with the results of other studies, which demonstrated the weakest seasonal correlation between air and soil temperature in the spring and summer. The authors noted that soil temperature is mainly influenced by factors unrelated to the diurnal cycle [46,51]. Furthermore, the authors found that the diurnal amplitude of soil temperatures and the dependence of soil temperature on air temperature decrease with soil profile depth [46,51]. This suggests that sand is less sensitive to temperature fluctuations with depth. In the present study, samples were collected from depths of 10 and 50 cm, which significantly reduced the influence of atmospheric conditions on the yeast population structure in the sand throughout the diurnal cycle.

Analyzing the yeast abundance at different depths, it was found that the obtained results are consistent with those reported by Grishkan and Kidron [52], who also observed higher average concentrations of yeast strains at a depth of 10 cm. This trend persisted across different research seasons. However, in the case of both parameters, the observed differences were not statistically significant. This observation may be linked to the higher content of organic carbon, water, and oxygen in the surface soil layers, where sand layers mix. Grishkan and Kidron’s [52] studies indicate decreased organic carbon content and oxygen availability with soil depth. The same study showed that this trend does not significantly change depending on the climate and that respiratory processes and biomass development of oxygen-affiliated organisms occur most intensively in the surface soil layers. Grishkan and Kidron’s [52] research suggests that the distribution of fungal communities in soil is related to the availability of organic matter, water, aeration, salinity, and UV radiation, emphasizing its complex and non-linear nature. Another factor influencing the distribution and abundance of microfungal consortia is soil and sand porosity [11,52]. Microbial biomass density reaches its maximum values in the surface soil layers (0.2–2 cm), with a second peak at a depth of 5–10 cm, followed by a sharp decline down to 50 cm [52]. This observation aligns with the present study.

During the research, differences in the taxonomic structure of soil yeast consortia depending on the soil profile depth were noted. The dominant part of the isolates obtained included species occurring at both depths (24 species; two genera). Species recorded in the number of three isolates or more were distinguished from this group. These were: *N. glabratus*, *C. jirovecii*, *C. moniliiforme*, *D. hansenii*, *Oosporidium margaritiferum*, and *Schwanniomyces polymorphus*. Based on these findings, it can be hypothesized that the mentioned species are insensitive to limited oxygen availability and are less susceptible to changes in oxygen content in the sand compared to other microorganisms. In the current study, 28 species and one genus were found exclusively at a depth of 10 cm, which may indicate their high oxygen affinity. At a depth of 50 cm, only 10 species and one genus were found, indicating their ability to grow in low-oxygen conditions. The taxonomic structure of the mycobiota at a depth of 50 cm was significantly poorer, showing a clear trend that yeasts can develop at various depths but are more commonly found in the surface soil layers. This is likely because most yeasts require oxygen for growth [53]. Nonetheless, other researchers suggest that yeasts may acquire the ability to develop in anaerobic conditions through horizontal gene transfer. It should be noted that other factors, such as soil structure (e.g., porosity), organic compound content (mainly carbon), water availability, UV radiation, salinity, and temperature, may also influence the species richness of yeasts in various soil layers [11,42,45,52].

Overall, more yeast strains were isolated from unsupervised beaches; however, a comparison of the number of isolates and yeast concentration in the sand did not reveal statistically significant differences. Similar results were obtained by other researchers [11,43]. The likely cause of this difference was the similar purpose of the beaches studied, as well as the cleaning and replenishment of sand on supervised beaches by municipal institutions, which may improve the sanitary condition of the sand. It should be noted, however, that the presence of municipal supervision at swimming beaches may encourage more people to use them, and the presence of users increases the quantitative and taxonomic structure of the mycobiota in the sand and water [10,43,45].

Among the most frequently reported yeasts on beaches are representatives of the genera: *Aureobasidium*, *Candida*, *Geotrichum*, *Exophiala*, *Metschnikowia*, *Rhodothorula*, and *Yarrowia* [14,42,43]. Yeasts from the genera *Candida*, *Cryptococcus*, and *Rhodotorula* are associated with sandy beaches [11]. In the present study, nearly 260 yeast strains were isolated from the sand of lake swimming beaches, among which 47 genera were recorded, including *Aureobasidium*, *Candida*, *Cryptococcus*, *Exophiala*, *Metschnikowia*, and *Rhodotorula*, confirming that beach sand is their natural ecological niche. Additionally, the literature indicates that the most commonly noted genera in lake waters are *Candida*, *Cryptococcus*, *Geotrichum*, *Hansenula*, *Pichia*, *Rhodotorula*, *Saccharomyces*, *Sporobolomyces*, and *Trichosporon* [10,11,31], and on the coastal phyllosphere, the genera *Candida*, *Cryptococcus*, *Debaryomyces*, *Metschnikowia*, *Pichia*, *Saccharomyces*, and *Rhodotorula* [9,10,11]. Five of the genera considered typical for bathing water and all those considered characteristic for coastal aquatic vegetation were isolated from the sand in this study. A summary of long-term studies conducted by Biedunkiewicz demonstrated the constant presence of over 40 yeast genera in lake waters in the Olsztyn region, and 16 associated with the aquatic vegetation found in their vicinity [10]. In the present study, 21 genera (48.8%) from those recorded by Biedunkiewicz and colleagues [10] in bathing water were noted, along with 13 genera (81.3%) that were isolated in their research from the phyllosphere. These observations indicate the continuous circulation of microfungi between the various components of the lake ecosystem, including during the washing of sand and vegetation by lake water, as well as through surface and subsurface runoff caused by precipitation. Beach users and animals living there, e.g., waterfowl, may also be an important vector of such transmission [10,11,42,45]. Numerous scientific papers indicate that species such as *B. californica*, *N. glabratus*, *C. moniliiforme*, *C. jirovecii*, *D. hansenii*, and *S. aeria* are soil microorganisms or are frequently isolated from soil, including beach sand [7,11,45,54,55]. The frequent notation of these species in the present study should therefore be considered a conventional observation.

A comparison of the taxonomic structure between the designated research seasons showed that 21 species and one genus (strain identified only to the genus level) were isolated in both seasons. Among this group, *B. californica*, *Nakaseomyces glabratus*, *C. moniliiforme*, *D. hansenii*, *Schwanniomyces polymorphus*, and *S aeria* were noted with three or more isolates in each season, suggesting that they are indigenous to the studied environments. This is supported by studies that classify these species as soil yeasts [7,11,19,56,57]. For the species *C. moniliiforme* and *D. hansenii*, the discrepancy in the number of isolates between seasons reached an order of magnitude. It can be hypothesized that these species may be more sensitive to seasonal changes in environmental parameters, such as weather conditions or beach user traffic and activity. This is corroborated by studies from other authors [7,10,11,13,21,45,55]. In the 2019 season, 29 species and two genera (strains identified only to the genus level) were recorded, while in 2020, 12 species and one genus were found. The probable cause of these observed differences is the limited traffic of beach users, a potential vector of microfungal transmission at bathing sites, due to the sanitary regulations related to the COVID-19 pandemic in 2020.

A total of 26 species and one genus (strains identified only to the genus level) were found on both supervised and unsupervised beaches. Among these, the species *B. californica*, *N. glabratus*, *Cutaneotrichosporon jirovecii*, *C. moniliiforme*, *D. hansenii*, and *S. aeria* were noted with three or more isolates on each type of beach. These species have previously been identified as characteristic or frequently isolated from soil and lake environments [7,10,11,45,54,55]. For the species *B. californica*, *N. glabratatus*, and *D. hansenii*, more isolates were found on supervised beaches. *N. glabratatus* often forms part of the physiological mycobiota of humans and, in immunocompromised individuals, can become pathogenic [10,11,13]. *B. californica* is classified as a soil yeast [7,19] and has also been isolated from water, plant debris, and animal droppings [58]. *B. californica* and *D. hansenii* are also found in food products [59,60]. The presence of these species suggests a link to human activity, as supervised beaches generally attract more visitors during the summer season. However, overall, half as many species were found exclusively on supervised beaches compared to those isolated solely from unsupervised beaches. This discrepancy is likely due to the better sanitary condition of supervised beaches, which are cleaned and have their sand replenished by supervisors.

The diurnal cycle was the parameter for which the greatest number of species were recorded in the sand, regardless of this parameter, compared to the other parameters adopted in the study (depth, season, type of supervision). Other researchers indicate that the taxonomic structure of yeasts in sand and soil generally changes due to seasonal dynamics rather than the diurnal cycle [11,46,51].

The taxonomic structure of yeast assemblages in sand was also compared between the beaches of different lakes. The most species were isolated from Lakes Ukiel and Skanda, and the fewest from Lake Tyrsko. The beaches of Ukiel and Skanda have the longest shorelines, the largest usable area, and are among the most frequently used for recreational purposes. The length of the shoreline increases the area where sand is washed by water, and beachgoers act as natural vectors of fungal transmission, promoting yeast circulation between the different components of the lake ecosystem [10,61]. The beach at Lake Tyrsko is managed by a private owner and is only accessible to guests of the nearby hotel, which significantly limits the number of users. Additionally, Lakes Ukiel, Kortowskie, and Skanda are eutrophic and exhibit high primary production, which is associated with high levels of nitrogen, oxygen, and phosphorus, and generally correlates with increased taxonomic diversity. In contrast, Lake Tyrsko, despite showing signs of aging, is characterized by low trophic status and poor water mineralization, which may limit the number of species transmitted to the sand [61].

Sixteen species and one genus were isolated from the beaches of more than two of the studied lakes. Among them, *C. moniliiforme*, *C. jirovecii*, and *D. hansenii* were isolated from the sand of all four studied lakes. Other studies confirm the presence of species belonging to these genera in lake water and beach sand [10,13,14,55]. These species can therefore be considered characteristic of the beach sand of the lakes studied and likely continuously associated with this type of ecological niche, which has sanitary and epidemiological significance.

All obtained yeast isolates were assigned BSL and RG index values in the context of their potential pathogenicity. Additionally, for the most frequently isolated species, a literature review was conducted regarding the infections they can cause.

Among the most frequently isolated species were *Cutaneotrichosporon jirovecii* and *C. moniliiforme.* These are potential human pathogens in immunocompromised individuals (Biedunkiewicz and Góralska [13]; Biedunkiewicz et al. [10]; Kulesza et al. [35]; Bałabański and Biedunkiewicz [8]). Studies indicate that species from the genus *Cutaneotrichosporon* can grow at human body temperature and produce hydrolytic enzymes that act as virulence factors promoting infections. Furthermore, the scientific literature indicates an increase in infections and resistance of *Cutaneotrichosporon* species to antifungal agents used by clinicians [62]. Researchers have noted that *C. jirovecii* can cause liver and skin damage, while *C. moniliiforme* tends to cause less invasive pathological changes [63]. The high risk these species pose to beachgoers is demonstrated by the fact that they were isolated from the sand of beaches at all the lakes studied.

The second most frequently noted species, *D. hansenii*, found on all the studied beaches, is associated with food products. It is generally considered saprotrophic, but there are reports in the literature of isolates from clinical material. Studies by Breuer and Harms [64] and Desnos-Ollivier et al. [59] showed that this species rarely causes infections such as bone infections, subcutaneous abscesses, keratitis, and allergic alveolitis in both immunocompetent and immunocompromised individuals.

During the study, numerous strains classified in the genus *Candida* were isolated. Species belonging to this genus are the most commonly isolated from the blood of hospitalized patients. Additionally, they are frequently found in the oral cavity, urinary tract, and female genital tract [65]. There is an increase in infections caused by *Candida* fungi in immunocompromised individuals, including cancer patients and postoperative patients [65]. Factors predisposing Candida species to infect humans include their ability to adhere to host epithelial cells and form biofilms [8,35,65,66,67]. In the present study, the dominant species was *Nakaseomyces glabratus* (formerly *C. glabrata*) with a single isolate of *C. albicans* also recorded. *N. glabratus* and *C. albicans* are among the four species responsible for approximately 95% of diagnosed candidiasis [65]. Both species are classified as BSL-2 and RG-2. They mainly cause infections of the skin and mucous membranes in immunocompromised individuals [10,13,43,56,65]. *N. glabratus* primarily causes infections of the oral cavity, urogenital system, and bloodstream, and it exhibits resistance to many antifungal agents [68]. *C. albicans* is considered an indicator of fecal contamination in the environment [11]. Among the frequently isolated species was also *Citeromyces matritensis*, the teleomorphic stage of *Candida globosa*. Our review of the literature did not reveal any reported clinical infections caused by this species.

Numerous strains of *B. californica* were isolated during the study This species is not pathogenic but rather saprotrophic [69]. Similarly, *Solicoccozyma aeria* was frequently noted. Research indicates that it can be isolated from animals, but there are no reports of human infections caused by this species [70]. *S. aeria* may be a part of the physiological mycobiota of the colon, as it has also been isolated from human feces [71]. Additionally, it has been linked to appetite suppression and the activation of interleukin 17 in sensitive individuals, which may be associated with disturbances in gut microbiota and mycobiota. Studies also show an increase in *S. aeria* expression in cancer patients, with the potential for considering this species as a marker of gastric cancer, while excluding it as an etiological factor in the disease [72]. In summary, the literature suggests that this species may have a positive impact on human health as a cancer marker. There are no reports of its potential pathogenicity to humans; however, its isolation from animals suggests it could be considered as a potential human pathogen.

Yeasts from the genus *Cryptococcus* were also noted in the beach sand. Many species from this genus exhibit pathogenic properties in both immunocompromised and immunocompetent individuals [73,74]. Fungi of the genus *Cryptococcus* cause cryptococcosis, which can affect the skin, lungs, and bloodstream, and, in advanced stages, lead to meningitis and brain edema [73,74]. The virulence factors of *Cryptococcus* include the production of polysaccharide capsules and high enzymatic activity (protease, urease, phospholipase) [73]. The only identified species, *C. amylolentus*, belongs to the non-pathogenic cryptococci, closely related to pathogenic species. This species has been shown to have infectious potential under induced conditions, but due to its sensitivity to thermal stress, it does not grow at human body temperature [73,74].

Six isolates from the genus *Exophiala* were recorded during the study. Of the identified species, *E. jeanselmei* was the most frequently isolated. This species can cause subcutaneous ulcers and nodules in transplant recipients [75]. In advanced cases, it leads to chronic, purulent infections of subcutaneous tissue [76]. The less frequently isolated species, *E. bergeri*, has been reported as an etiological agent of nail infections [77], while *E. castellanii* has been noted in subcutaneous infections [78]. All species from the genus *Exophiala* isolated in this study are potential pathogens for immunocompromised individuals, as they are classified as BSL-2 and RG-2 [8,10,13,35,78].

All remaining species were isolated in numbers of six or fewer strains. Due to their rare (episodic) occurrence in the studied environments and simultaneous classification as BSL-1 and RG-1, it was assumed that they do not pose a potential threat to beach users. This risk assessment is consistent with the approach outlined in the 2021 WHO guidelines on recreational water quality, which emphasize prioritizing microbiological monitoring efforts toward organisms with higher pathogenic potential and relevance to public health [1].

## 5. Conclusions

The examination of the sanitary condition of beach sand at supervised and unsupervised swimming areas near Lakes Kortowskie, Skanda, Tyrsko, and Ukiel revealed the presence of species classified as potential human pathogens. Particular attention should be given to *N. glabratus*, *Cutaneotrichosporon jirovecii*, and *C. moniliiforme*, which were among the five most frequently isolated species, exhibited a cosmopolitan character in relation to the ecological niches studied, and were classified as Biosafety Level 2 (BSL-2) and Risk Group 2 (RG-2). The research demonstrated that beach sand at swimming areas may pose a potential health risk for individuals with temporary or chronic immunocompromised conditions. In this context, it is essential to conduct continuous sanitary-epidemiological monitoring of beach sand and implement preventive measures to mitigate the risks associated with the presence of potentially pathogenic yeasts in the sand, such as regular sand cleaning and replenishment. The present study, combined with the literature data, revealed that knowledge regarding the ecological and taxonomic characteristics of yeast populations in beach sand at swimming areas is significantly limited. Furthermore, there is a lack of legal regulations defining reference standards for the mycological cleanliness of such sand. In light of this, the introduction of such standards and the supervision of beach sand at swimming areas by institutions authorized to conduct sanitary inspections is recommended, along with further research in this field.

## Figures and Tables

**Table 1:** List of species isolated in the study, including division, BSL and RG indicator values, and number of isolates obtained, categorized by research parameters.

No.	Species	MI	DV	BSL	RG	SUM	M	E	d10	d50	S	US	s19	s20	LK	LS	LT	LU
1	*Aureobasidium pullulans* (de Bary & Löwenthal) G. Arnaud 1918	+	A	1	1	5	4	1	2	3	1	4	1	4	1	0	3	1
2	*Barnettozyma californica* (Lodder) Kurtzman, Robnett & Bas.-Powers 2008	+	A	1	1	12	7	5	11	1	8	4	8	4	2	0	1	9
3	** *Candida albicans* (C.P. Robin) Berkhout 1923		A	2	2	1	1	0	1	0	1	0	0	1	0	1	0	0
4	*Citeromyces matritensis* (Santa María) Santa María 1957		A	1	1	8	5	3	8	0	6	2	2	6	2	5	0	1
5	*Clavispora lusitaniae* Rodr. Mir. 1979 (anamorfa: *Candida lusitaniae*)		A	2	2	1	1	0	0	1	1	0	0	1	0	0	0	1
6	*Cryptococcus amylolentus* (Van der Walt, D.B. Scott & Klift) Golubev 1981		B	1	1	4	1	3	3	1	0	4	0	4	3	0	1	0
7	*Cryptococcus uniguttulatus* (Wolfram & Zach) Phaff & Fell 1970		B	1	1	1	1	0	1	0	0	1	0	1	0	1	0	0
8	*Cutaneotrichosporon jirovecii* (Frágner) Xin Zhan Liu, F.Y. Bai, M. Groenew. & Boekhout 2015	+	B	2	2	16	8	8	12	4	4	12	15	1	4	6	1	5
9	*Cutaneotrichosporon moniliiforme* (E. Guého & M.T. Sm.) Xin Zhan Liu, F.Y. Bai, M. Groenew. & Boekhout 2015	+	B	2	2	28	18	10	17	11	6	22	25	3	8	9	3	8
10	*Cyniclomyces guttulatus* (C.P. Robin) Van der Walt & D.B. Scott 1971		A	1	1	2	2	0	0	2	1	1	0	2	0	0	1	1
11	** *Debaryomyces hansenii* (Zopf) Lodder & Kreger-van Rij 1984	+	A	1	1	24	7	17	10	14	13	11	5	19	4	7	2	11
12	*Dothiora sorbi* (Wahlenb.) Fuckel 1870	+	A	1	1	1	0	1	0	1	1	0	0	1	0	0	0	1
13	*Exophiala bergeri* Haase & de Hoog 1999		A	2	2	1	0	1	0	1	0	1	0	1	0	0	1	0
14	*Exophiala castellanii* Iwatsu, Nishim. & Miyaji 1984		A	2	2	2	1	1	2	0	0	2	0	2	0	0	2	0
15	*Exophiala jeanselmei* (Langeron) McGinnis & A.A. Padhye 1977		A	2	2	3	3	0	0	3	3	0	0	3	0	3	0	0
16	** *Geotrichum albidum* (Lagerh.) H.Y. Zhu, X.Z. Liu & F.Y. Bai 2024		A	1	1	3	2	1	3	0	2	1	0	3	1	2	0	0
17	*Geotrichum galactomycetum* H.Y. Zhu, X.Z. Liu & F.Y. Bai 2024		A	1	1	2	1	1	1	1	2	0	0	2	0	1	0	1
18	*Hanseniaspora osmophila* (Niehaus) Phaff, M.W. Mill. & Shifrine 1956		A	1	1	1	1	0	1	0	0	1	1	0	0	0	1	0
19	*Isabelozyma rhagii* (Diddens & Lodder) Q.M. Wang, Yurkov, Boekhout & F.Y. Bai 2024		A	1	1	1	1	0	1	0	0	1	1	0	1	0	0	0
20	*Komagataella pastoris* (Guillierm.) Y. Yamada, M. Matsuda, K. Maeda & Mikata 1995		A	1	1	3	2	1	2	1	2	1	1	2	0	1	0	2
21	*Kondoa malvinella* (Fell & I.L. Hunter) Y. Yamada, Nakagawa & I. Banno 1989		B	1	1	4	2	2	2	2	2	2	2	2	0	2	2	0
22	*Kregervanrija fluxuum* (Phaff & E.P. Knapp) Kurtzman 2006 (anamorfa: *Candida vini*)		A	1	1	1	1	0	1	0	1	0	0	1	0	1	0	0
23	*Leucosporidium scottii* Fell, Statzell, I.L. Hunter & Phaff 1970		B	1	1	1	0	1	1	0	0	1	0	1	0	0	0	1
24	*Lipomyces lipofer* (Den Dooren) Lodder & Kreger-van Rij 1952	+	A	1	1	2	2	0	2	0	2	0	0	2	0	0	0	2
25	*Lodderomyces elongisporus* (Recca & Mrak) Van der Walt 1971		A	1	1	1	0	1	1	0	1	0	0	1	0	0	0	1
26	*Metschnikowia pulcherrima* Pitt & M.W. Mill. 1968		A	1	1	1	1	0	1	0	0	1	1	0	0	1	0	0
27	*Moniliella spathulata* (de Hoog) C.A. Rosa & Lachance 2009		B	1	1	2	1	1	2	0	0	2	0	2	2	0	0	0
28	*Mycogloea nipponica* Bandoni 1998		B	1	1	6	2	4	4	2	4	2	0	6	1	3	0	2
29	** *Nadsonia commutata* Golubev 1973		A	1	1	3	3	0	1	2	1	2	2	1	0	0	0	3
30	*Nadsonia fulvescens var. elongata* (Konok.) Golubev & M.T. Sm. 1989		A	1	1	5	3	2	3	2	0	5	4	1	3	2	0	0
31	*Naganishia albida* (Saito) Xin Zhan Liu, F.Y. Bai, M. Groenew. & Boekhout 2015		B	1	1	3	1	2	3	0	2	1	1	2	0	1	0	2
32	** *Nakaseomyces glabratus* (H.W. Anderson) Sugita & M. Takash 2022		A	2	2	12	6	6	8	4	7	5	3	9	0	5	1	6
33	*Octosporomyces octosporus* (Beij.) Kudryavtsev 1960		A	1	1	1	0	1	1	0	0	1	1	0	1	0	0	0
34	*Ogataea angusta* (Teun., H.H. Hall & Wick.) S.O. Suh & J.J. Zhou 2010		A	1	1	2	1	1	2	0	0	2	2	0	2	0	0	0
35	*Ogataea minuta* (Wick.) Y. Yamada, K. Maeda & Mikata 1994		A	1	1	1	0	1	1	0	0	1	0	1	0	1	0	0
36	*Oosporidium margaritiferum* Stautz 1931		A	1	1	6	5	1	3	3	1	5	0	6	2	3	0	1
37	*Papiliotrema laurentii* (Kuff.) Xin Zhan Liu, F.Y. Bai, M. Groenew. & Boekhout 2015	+	B	1	1	4	3	1	3	1	2	2	0	4	0	2	0	2
38	*Papiliotrema pseudoalba* (Nakase & M. Suzuki) Xin Zhan Liu, F.Y. Bai, M. Groenew. & Boekhout 2015)		B	1	1	3	3	0	0	3	0	3	0	3	3	0	0	0
39	** *Pichia fermentans* Lodder 1932		A	1	1	1	0	1	1	0	1	0	1	0	0	0	0	1
40	*Pichia membranifaciens* (E.C. Hansen) E.C. Hansen 1904		A	1	1	6	4	2	5	1	2	4	4	2	0	5	0	1
41	*Pichia pseudolambica* (M.T. Sm. & Poot) H.Y. Zhu, X.Z. Liu & F.Y. Bai 2024	+	A	1	1	5	4	1	3	2	0	5	1	4	0	3	0	2
42	*Pichia terricola* Van der Walt 1957		A	1	1	1	0	1	1	0	1	0	0	1	0	0	0	1
43	*Rhodotorula diobovata* (S.Y. Newell & I.L. Hunter) Q.M. Wang, F.Y. Bai, M. Groenew. & Boekhout 2015		B	1	1	1	1	0	1	0	0	1	0	1	1	0	0	0
44	*Saccharomyces bayanus* Sacc. 1895		A	1	1	3	1	2	0	3	2	1	0	3	1	2	0	0
45	*Saccharomyces cerevisiae* (Desm.) Meyen 1838		A	1	1	5	1	4	4	1	1	4	1	4	0	3	1	1
46	*Saccharomyces mikatae* G.I. Naumov, S.A. James, E.S. Naumova, E.J. Louis & I.N. Roberts 2000		A	1	1	1	1	0	0	1	0	1	0	1	0	0	1	0
47	** *Saccharomycodes ludwigii* (E.C. Hansen) E.C. Hansen 1904		A	1	1	3	2	1	3	0	1	2	1	2	1	1	1	0
48	*Saitozyma podzolica* (Babeva & Reshetova) Xin Zhan Liu, F.Y. Bai, M. Groenew. & Boekhout 2015	+	B	1	1	3	2	1	1	2	2	1	1	2	0	1	0	2
49	*Schizosaccharomyces pombe* Lindner 1893		A	1	1	1	1	0	1	0	0	1	1	0	0	0	1	0
50	*Schwanniomyces capriottii* M. Suzuki & Kurtzman 2010	+	A	1	1	5	4	1	3	2	2	3	5	0	2	0	1	2
51	*Schwanniomyces occidentalis* Klöcker 1909		A	1	1	2	0	2	2	0	0	2	2	0	2	0	0	0
52	** *Schwanniomyces polymorphus* (Klöcker) M. Suzuki & Kurtzman 2010		A	1	1	8	4	4	5	3	2	6	3	5	0	4	2	2
53	*Schwanniomyces vanrijiae* (Van der Walt & Tscheuschner) M. Suzuki & Kurtzman 2010		A	1	1	1	0	1	1	0	0	1	1	0	1	0	0	0
54	*Solicoccozyma aeria* (Saito) Yurkov 2015	+	B	1	1	11	7	4	10	1	3	8	3	8	7	1	0	3
55	Sporobolomyces xanthus (Nakase, G. Okada & Sugiy.) Boekhout 1991		B	1	1	1	1	0	1	0	0	1	0	1	0	0	0	1
56	*Sydowia polyspora* (Bref. & Tavel) E. Müll. 1953		A	1	1	1	0	1	1	0	1	0	0	1	0	1	0	0
57	*Tausonia pullulans* (Lindner) Xin Zhan Liu, F.Y. Bai, M. Groenew. & Boekhout 2015	+	B	1	1	6	2	4	5	1	1	5	6	0	2	2	0	2
58	*Thelebolus globosus* Brumm. & de Hoog 2005	+	A	1	1	2	2	0	0	2	0	2	0	2	2	0	0	0
59	*Torulaspora globosa* (Klöcker) Van der Walt & Johannsen 1975		A	1	1	4	2	2	4	0	1	3	2	2	0	3	0	1
60	*Vanderwaltozyma polyspora* (Van der Walt) Kurtzman 2003		A	1	1	5	3	2	3	2	0	5	3	2	1	3	1	0
61	*Vanrija humicola* (Dasz.) R.T. Moore 1980		B	1	1	1	0	1	0	1	0	1	1	0	1	0	0	0
62	*Wickerhamomyces anomalus* (E.C. Hansen) Kurtzman, Robnett & Bas.-Powers 2008 (anamorfa: Candida peliculosa)		A	1	1	1	0	1	1	0	1	0	0	1	0	0	0	1
63	*Bullera* sp.		B	1	1	2	1	1	1	1	2	0	0	2	0	0	0	2
64	* *Cryptococcus* sp.	+	B	1/2	1/2	5	3	2	3	2	2	3	3	2	2	1	0	2
65	*Dipodascus* sp.		A	1	1	1	1	0	1	0	0	1	0	1	0	0	0	1
66	*Kluyveromyces* sp.		A	1	1	1	0	1	0	1	1	0	1	0	0	0	0	1
	Sum *		-	-	-	264	147	117	175	89	100	164	115	149	63	87	27	87
No.	**SPECIES**	**MI**	**DV**	**BSL**	**RG**	**SUM**	**M**	**E**	**d10**	**d50**	**S**	**US**	**s19**	**s20**	**LK**	**LS**	**LT**	**LU**

Number of isolates—how many times a given species was isolated during the study, and a pure culture was obtained on Sabouraud agar, and safely preserved for storage. e.g., *Candida albicans*—species classified to biosafety level 2 (BSL-2). ** e.g., *Saccharomycodes ludwigii*—species included in two-species isolates. e.g., * *Cryptococcus* sp.—BSL index value varies depending on species. **SUM**—total number of isolates of a given species. Sum *—total number of isolates (species of two-species isolates were counted separately and included in total sum of isolates). **MI**—“+” indicates that the species was confirmed using molecular identification methods (consistent with results obtained from phenotypic identification). **DV**—division (A—*Ascomycota*; B—*Basidiomycota*). **BSL**—Biosafety Level. **RG**—Risk Group. **M**—number of isolates obtained in the morning. **E**—number of isolates obtained in the evening. **d10**—number of isolates obtained from sand collected from a depth of 10 cm. **d50**—number of isolates obtained from sand collected from a depth of 50 cm. **S**—number of isolates obtained from sand of supervised beaches. **US**—number of isolates obtained from sand of unsupervised beaches. **s19**—number of isolates obtained in the 2019 research season. **s20**—number of isolates obtained in the 2020 research season. **LK**—number of isolates obtained from the beach of Lake Kortowskie. **LS**—number of isolates obtained from the beaches of Lake Skanda. **LT**—number of isolates obtained from the beach of Lake Tyrsko. **LU**—number of isolates obtained from the beach of Lake Ukiel.

**Table 2:** List of two-species isolates.

No.	Species	Research Season	Lake
1	*Nakaseomyces glabratus* (H.W. Anderson) Sugita & M. Takash 2022 *Pichia fermentans* Lodder 1932	2019	Ukiel
2	*Debaryomyces hansenii* (Zopf) Lodder & Kreger-van Rij 1984 *Nadsonia commutata* Golubev 1973	2019	Ukiel
3	*Debaryomyces hansenii* (Zopf) Lodder & Kreger-van Rij 1984 *Nadsonia commutata* Golubev 1973	2019	Ukiel
4	*Candida albicans* (C.P. Robin) Berkhout 1923 *Geotrichum albidum* (Lagerh.) H.Y. Zhu, X.Z. Liu & F.Y. Bai 2024	2020	Skanda
5	*Saccharomycodes ludwigii* (E.C. Hansen) E.C. Hansen 1904 *Schwanniomyces polymorphus* (Klöcker) M. Suzuki & Kurtzman 2010	2020	Tyrsko

**Table 3:** Statistical dependence of the number of isolates and strain concentration on meteorological parameters (Spearman rank correlation coefficient).

Parameter	Isolates Number	Average Strains Concentration
**Temperature**	Independent of the parameter (*R* = 0.2991; *p* = 0.1355)	* Depends on temperature (*R* = 0.4339; *p* = 0.0210)
**Air humidity**	Independent of the parameter (*R* = −0.0837; *p* = 0.6716)	Independent of the parameter (*R* = −0.0766; *p* = 0.6984)
**UV index**	Independent of the parameter (*R* = 0.1056; *p* = 0.5926)	Independent of the parameter (*R* = 0.3396; *p* = 0.0770)

* statistically significant results (*p* < 0.05).

## Data Availability

Sequences of ITS regions have been deposited in the GenBank database. Accession number range: PQ882533–PQ882625.

## Supplementary Materials

The following supporting information can be downloaded at: https://www.mdpi.com/article/10.3390/pathogens14080744/s1; Table S1. The number of isolated yeasts.

## Abbreviations

BSL—biosafety level; CFU—colony-forming unit; PCR—polymerase chain reaction; RBC—Rose Bengal Chloramphenicol Agar, RG—risk group; WHO—World Health Organization.

## Appendix A. Values of Meteorological Parameters During Sand Sampling

**SAMPLING PERIOD**	**TEMPERATURE**		**AVERAGE TEMPERATURE**	**AIR HUMIDITY**		**UV INDEX**
	**MORNING**	**EVENING**		**MORNING**	**EVENING**	
**APRIL/2019**	6 °C	14 °C	D 12 °C N 5 °C	36%	32%	3/10
**MAY/2019**	8 °C	15 °C	D 15 °C N 8 °C	73%	29%	4/10
**JUNE/2019**	17 °C	25 °C	D 25 °C N 15 °C	64%	45%	7/10
**JULY/2019**	20 °C	21 °C	D 24 °C N 15 °C	75%	70%	6/10
**AUGUST/2019**	16 °C	19 °C	D 22 °C N 14 °C	85%	51%	5/10
**SEPTEMBER/2019**	13 °C	16 °C	D 18 °C N 11 °C	75%	51%	4/10
**OCTOBER/2019**	12 °C	9 °C	D 13 °C N 7 °C	91%	91%	2/10
**APRIL/2020**	4 °C	12 °C	D 13 °C N 5 °C	69%	29%	3/10
**MAY/2020**	6 °C	6 °C	D 17 °C N 9 °C	93%	94%	4/10
**JUNE/2020**	12 °C	18 °C	D 21 °C N 12 °C	64%	42%	6/10
**JULY/2020**	19 °C	23 °C	D 22 °C N 14 °C	85%	59%	6/10
**AUGUST/2020**	19 °C	24 °C	D 23 °C N 15 °C	39%	39%	5/10
**SEPTEMBER/2020**	13 °C	16 °C	D 18 °C N 11 °C	92%	90%	0/10
**OCTOBER/2020**	11 °C	8 °C	D 13 °C N 6 °C	88%	88%	2/10
D—average temperature of the month during the day, N—average temperature of the month at night.						

## Appendix B. Estimated Number of Users of the “Ukiel” Recreational Complex and Supervised Beach on Lake Skanda During the Year—Data of the Sports and Recreation Center in Olsztyn

**LAKE UKIEL**				
**MONTH**	**2019**		**2020**	
	**SUM OF ENTRIES**	**SUM OF EXITS**	**SUM OF ENTRIES**	**SUM OF EXITS**
**JANUARY**	32,751	29,601	14,557	13,889
**FEBRUARY**	64,786	84,381	4015	3385
**MARCH**	124,985	133,036	3458	3094
**APRIL**	106,946	126,388	1562	1918
**MAY**	142,397	132,035	8416	9846
**JUNE**	110,436	108,515	4343	5640
**JULY**	174,234	160,329	THE SUPERVISOR DID NOT KEEP A REGISTER DUE TO THE COVID-19 PANDEMIC	
**AUGUST**	72,885	72,431		
**SEPTEMBER**	53,825	57,711		
**OCTOBER**	24,177	27,222		
**NOVEMBER**	12,461	13,223		
**DECEMBER**	10,828	11,499		
**SUM**	**930,711**	**956,371**	**36,351**	**37,772**
**LAKE SKANDA**				
**MONTH**	**2019**		**2020**	
	**PEOPLE PER DAY**	**MONTHLY**	**PEOPLE PER DAY**	**MONTHLY**
**JANUARY**	THE SUPERVISORY DOES NOT KEEP A REGISTER (OFF-SEASON PERIOD)			
**FEBRUARY**				
**MARCH**				
**APRIL**				
**MAY**				
**JUNE**	Approx. 150 (weekends 500)	Approx. 3600	Approx. 150 (weekends 500)	Approx. 3300
**JULY**		Approx. 9300		Approx. 10,200
**AUGUST**		Approx. 10,200		Approx. 9000
**SEPTEMBER**		Approx. 2100		Approx. 1950
**OCTOBER**	THE SUPERVISORY DOES NOT KEEP A REGISTER (OFF-SEASON PERIOD)			
**NOVEMBER**				
**DECEMBER**				
**SUM**	**-**	**Approx. 25,200**	**-**	**Approx. 24,450**
