# Supplementary material for: Yeast Diversity on Sandy Lake Beaches Used for Recreation in Olsztyn, Poland
Source: Pathogens. 2025 Jul 29;14(8):744. doi: 10.3390/pathogens14080744 (PMC12389204; doi:10.3390/pathogens14080744)
Supplement: Supplementary file 1 [file pathogens-14-00744-s001.zip › pathogens-3746109-supplementary.pdf]

Supplementary Table S1. The number of isolated yeasts

SEASON 2019 (112)							
**Isolate number**	**Sampling code**	**Number of morphologically identical colonies**	**Number of yeast (cfu/cm^3)**	**Average number of yeast at a depth of 10 cm from the beaches of a given lake (cfu/cm^3)**	**Average number of yeast at a depth of 50 cm from the beaches of a given lake (cfu/cm^3)**	**Average number of yeast at both depths of 50 cm from the beaches of a given lake (cfu/cm^3)**	**Month (number of isolates)**
**1**	r/04.04.19/T/NN/10	**1**	1,00E+02	1,00E+02	0.00E+00	1,00E+02	**APRIL (23)**
**2**	w/04.04.19/T/NN/10	**1**	1,00E+02				
**3**	r/04.04.19/K/NN/10	**1**	1,00E+02	1,60E+02	0.00E+00	8.00E+01	
**4**	r/04.04.19/K/NN/10	**2**	2,00E+02				
**5**	r/04.04.19/K/NN/10	**2**	2,00E+02				
**6**	w/04.04.19/K/NN/10	**2**	2,00E+02				
**7**	w/04.04.19/K/NN/10	**1**	1,00E+02				
**8**	r/04.04.19/S/NN/10	**1**	1,00E+02	1,00E+02	1,67E+02	1,33E+02	
**9**	r/04.04.19/S/N/10	**1**	1,00E+02				
**10**	r/04.04.19/S/N/50	**2**	2,00E+02				
**11**	r/04.04.19/S/N/50	**1**	1,00E+02				
**12**	w/04.04.19/S/N/10	**1**	1,00E+02				
**13**	w/04.04.19/S/N/50	**2**	2,00E+02				
**14**	r/04.04.19/U/NN/10	**1**	1,00E+02	1,43E+02	2,00E+02	1,71E+02	
**15**	w/04.04.19/U/NN/50	**1**	1,00E+02				
**16**	r/04.04.19/UM/N/10	**1**	1,00E+02				
**17**	r/04.04.19/UO/N/10	**1**	1,00E+02				
**18**	w/04.04.10/UO/N/50	**1**	1,00E+02				
**19**	r/04.04.19/US/N/10	**4**	4,00E+02				
**20**	w/04.04.19/US/N/10	**1**	1,00E+02				
**21**	w/04.04.19/US/N/10	**1**	1,00E+02				
**22**	w/04.04.19/US/N/10	**1**	1,00E+02				
**23**	w/04.04.19/US/N/50	**4**	4,00E+02				
**24**	r/01.05.19/T/NN/10	**2**	2,00E+02	2.00E+02	1.00E+02	1.50E+02	**MAY (11)**
**25**	r/01.05.19/T/NN/50	**1**	1,00E+02				
**26**	r/01.05.19/K/NN/10	**1**	1,00E+02	1.00E+02	0.00E+00	5.00E+01	
**27**	r/01.05.19/K/NN/10	**1**	1,00E+02				
**28**	r/01.05.19/S/NN/10	**3**	3,00E+02	2,14E+02	0,00E+00	1,07E+02	
**29**	r/01.05.19/S/NN/10	**2**	2,00E+02				
**30**	r/01.05.19/S/NN/10	**5**	5,00E+02				
**31**	r/01.05.19/S/NN/10	**2**	2,00E+02				
**32**	r/01.05.19/S/NN/10	**1**	1,00E+02				
**33**	w/01.05.19/S/NN/10	**1**	1,00E+02				
**34**	w/01.05.19/UO/N/10	**1**	1,00E+02				
**35**	w/03.06.19/T/NN/10	**1**	1,00E+02	1.00E+02	0.00E+00	5.00E+01	**JUNE (1**

**36**	r/03.06.19/K/NN/10	**3**	3,00E+02	4,75E+02	1,00E+03	7,38E+02	
**37**	r/03.06.19/K/NN/10	**2**	2,00E+02				
**38**	r/03.06.19/K/NN/50	**18**	1,80E+03				
**39**	r/03.06.19/K/NN/50	**11**	1,10E+03				
**40**	w/03.06.19/K/NN/10	**5**	5,00E+02				
**41**	w/03.06.19/K/NN/10	**9**	9,00E+02				
**42**	w/03.06.19/K/NN/50	**1**	1,00E+02				
**43**	r/03.06.19/S/NN/50	**11**	1,10E+03	0,00E+00	3,75E+02	1,88E+02	
**44**	r/03.06.19/S/NN/50	**2**	2,00E+02				
**45**	r/03.06.19/S/NN/50	**1**	1,00E+02				
**46**	w/03.06.19/S/N/50	**1**	1,00E+02				
**47**	r/03.06.19/U/NN/10	**1**	1,00E+02	1,33E+02	1,37E+03	7,50E+02	
**48**	r/03.06.19/U/NN/50	**23**	2,30E+03				
**49**	r/03.06.19/U/NN/50	**5**	5,00E+02				
**50**	w/03.06.19/UO/N/10	**2**	2,00E+02				
**51**	w/03.06.19/US/N/10	**1**	1,00E+02				
**52**	w/03.06.19/US/N/50	**13**	1,30E+03				
**53**	r/01.07.19/K/NN/10	**4**	4,00E+02	7,00E+02	5,20E+03	2,95E+03	**JULY (19)**
**54**	r/01.07.19/K/NN/50	**52**	5,20E+03				
**55**	w/01.07.19/K/NN/10	**9**	9,00E+02				
**56**	w/01.07.19/K/NN/10	**7**	7,00E+02				
**57**	w/01.07.19/K/NN/10	**8**	8,00E+02				
**58**	r/01.07.19/S/NN/10	**1**	1,00E+02	1,87E+04	1,00E+02	9,41E+03	
**59**	r/01.07.19/S/NN/10	**5**	5,00E+02				
**60**	w/01.07.19/S/NN/10	**484**	4,84E+04				
**61**	w/01.07.19/S/NN/10	**15**	1,50E+03				
**62**	w/01.07.19/S/NN/10	**431**	4,31E+04				
**63**	w/01.07.19/S/N/50	**1**	1,00E+02				
**64**	r/01.07.19/U/NN/10	**3**	3,00E+02	3,00E+02	0,00E+00	1,50E+02	
**65**	r/01.07.19/U/NN/10	**1**	1,00E+02				
**66**	r/01.07.19/U/NN/10	**1**	1,00E+02				
**67**	r/01.07.19/UO/N/10	**6**	6,00E+02				
**68**	r/01.07.19/UO/N/10	**1**	1,00E+02				
**69**	r/01.07.19/UO/N/10	**1**	1,00E+02				
**70**	w/01.07.19/UO/N/10	**6**	6,00E+02				
**71**	w/01.07.19/UO/N/10	**5**	5,00E+02				
**72**	r/04.08.19/T/NN/10	**1**	1,00E+02	1,00E+02	1,00E+02	1,00E+02	**AUGUST (11)**
**73**	w/04.08.19/T/NN/50	**1**	1,00E+02				
**74**	r/04.08.19/K/NN/10	**1**	1,00E+02	1.00E+02	0.00E+00	5.00E+01	
**75**	w/04.08.19/K/NN/10	**1**	1,00E+02				
**76**	w/04.08.19/S/NN/10	**2**	2,00E+02	1,50E+02	0,00E+00	7,50E+01	
**77**	w/04.08.19/S/NN/50	**1**	1,00E+02				
**78**	w/04.08.19/U/NN/50	**4**	4,00E+02	2,50E+02	4,33E+02	3,42E+02	
**79**	r/04.08.19/UM/N/50	**1**	1,00E+02				
**80**	w/04.08.19/UM/N/50	**8**	8,00E+02				
**81**	w/04.08.19/UO/N/10	**2**	2,00E+02				
**82**	r/04.08.19/US/N/10	**3**	3,00E+02				
**83**	r/06.09.19/T/NN/10	**1**	1,00E+02	1.00E+02	0.00E+00	5.00E+01	**SEPTEMBER (15)**
**84**	r/06.09.19/K/NN/10	**1**	1,00E+02	1,00E+02	1,50E+02	1,25E+02	
**85**	r/06.09.19/K/NN/50	**1**	1,00E+02				
**86**	r/06.09.19/K/NN/50	**3**	3,00E+02				
**87**	r/06.09.19/K/NN/50	**1**	1,00E+02				
**88**	w/06.09.19/K/NN/10	**1**	1,00E+02				

**89**	w/06.09.19/K/NN/10	**1**	1,00E+02				
**90**	w/06.09.19/K/NN/50	**1**	1,00E+02				
**91**	r/06.09.19/S/NN/10	**1**	1,00E+02	1,71E+02	0,00E+00	8,57E+01	
**92**	r/06.09.19/S/NN/10	**5**	5,00E+02				
**93**	r/06.09.19/S/NN/10	**1**	1,00E+02				
**94**	r/06.09.19/S/NN/10	**1**	1,00E+02				
**95**	w/06.09.19/S/NN/10	**2**	2,00E+02				
**96**	r/06.09.19/S/N/10	**1**	1,00E+02				
**97**	r/06.09.19/UM/N/10	**1**	1,00E+02				
**98**	r/02.10.19/T/NN/10	**1**	1,00E+02	1,67E+02	0,00E+00	8,33E+01	**OCTOBER (15)**
**99**	r/02.10.19/T/NN/10	**3**	3,00E+02				
**100**	r/02.10.19/T/NN/10	**1**	1,00E+02				
**101**	r/02.10.19/K/NN/50	**1**	1,00E+02	1,67E+02	1,00E+02	1,33E+02	
**102**	w/02.10.19/K/NN/10	**2**	2,00E+02				
**103**	w/02.10.19/K/NN/10	**1**	1,00E+02				
**104**	w/02.10.19/K/NN/10	**2**	2,00E+02				
**105**	w/02.10.19/K/NN/50	**1**	1,00E+02				
**106**	r/02.10.19/S/NN/10	**1**	1,00E+02	1,40E+02	1,00E+02	1,20E+02	
**107**	r/02.10.19/S/NN/10	**1**	1,00E+02				
**108**	r/02.10.19/S/NN/10	**2**	2,00E+02				
**109**	w/02.10.19/S/NN/10	**1**	1,00E+02				
**110**	w/02.10.19/S/NN/10	**2**	2,00E+02				
**111**	w/02.10.19/S/NN/50	**1**	1,00E+02				
**112**	r/02.10.19/UM/N/10	**1**	1,00E+02	1.00E+02	0.00E+00	5.00E+01	
**SEASON 2020 (147)**							
**113**	r/05.04.2020/K/NN/10	**2**	2,00E+02	1,33E+02	1,20E+02	1,27E+02	**APRIL (23)**
**114**	r/05.04.2020/K/NN/10	**1**	1,00E+02				
**115**	r/05.04.2020/K/NN/50	**2**	2,00E+02				
**116**	r/05.04.2020/K/NN/50	**1**	1,00E+02				
**117**	r/05.04.2020/K/NN/50	**1**	1,00E+02				
**118**	r/05.04.2020/K/NN/50	**1**	1,00E+02				
**119**	r/05.04.2020/K/NN/50	**1**	1,00E+02				
**120**	w/05.04.2020/K/NN/10	**1**	1,00E+02				
**121**	r/05.04.2020/S/NN/10	**1**	1,00E+02	4,14E+02	1,00E+02	2,57E+02	
**122**	r/05.04.2020/S/NN/10	**1**	1,00E+02				
**123**	r/05.04.2020/S/NN/50	**1**	1,00E+02				
**124**	w/05.04.2020/S/NN/10	**1**	1,00E+02				
**125**	w/05.04.2020/S/NN/10	**2**	2,00E+02				
**126**	w/05.04.2020/S/NN/10	**19**	1,90E+03				
**127**	w/05.04.2020/S/NN/10	**1**	1,00E+02				
**128**	w/05.04.2020/S/NN/10	**4**	4,00E+02				
**129**	r/05.04.2020/S/N/50	**1**	1,00E+02				
**130**	r/05.04.2020/S/N/50	**1**	1,00E+02				
**131**	r/05.04.2020/S/N/50	**1**	1,00E+02				
**132**	w/05.04.2020/S/N/50	**1**	1,00E+02				
**133**	r/05.04.2020/U/NN/10	**1**	1,00E+02	1,00E+02	1,00E+02	1,00E+02	
**134**	r/05.04.2020/US/N/10	**1**	1,00E+02				
**135**	r/05.04.2020/US/N/50	**1**	1,00E+02				
**136**	r/05.05.2020/T/NN/50	**1**	1,00E+02	0.00E+00	1.00E+02	5.00E+01	**MAY (21)**
**137**	r/05.05.2020/K/NN/50	**2**	2,00E+02	1,00E+02	1,25E+02	1,13E+02	
**138**	r/05.05.2020/K/NN/50	**1**	1,00E+02				
**139**	w/05.05.2020/K/NN/10	**1**	1,00E+02				
**140**	w/05.05.2020/K/NN/50	**1**	1,00E+02				

**141**	w/05.05.2020/K/NN/50	**1**	1,00E+02				
**142**	r/05.05.2020/S/NN/10	**7**	7,00E+02	2,75E+02	1,00E+02	1,88E+02	
**143**	r/05.05.2020/S/N/10	**1**	1,00E+02				
**144**	r/05.05.2020/S/N/10	**1**	1,00E+02				
**145**	r/05.05.2020/S/N/50	**1**	1,00E+02				
**146**	w/05.05.2020/S/N/10	**2**	2,00E+02				
**147**	w/05.05.2020/S/N/50	**1**	1,00E+02				
**148**	r/05.05.2020/UM/N/10	**1**	1,00E+02	1,40E+02	2,50E+02	1,95E+02	
**149**	r/05.05.2020/UM/N/50	**1**	1,00E+02				
**150**	r/05.05.2020/UM/N/50	**1**	1,00E+02				
**151**	r/05.05.2020/UM/N/50	**1**	1,00E+02				
**152**	w/05.05.2020/UO/N/10	**1**	1,00E+02				
**153**	w/05.05.2020/UO/N/10	**1**	1,00E+02				
**154**	w/05.05.2020/UO/N/10	**1**	1,00E+02				
**155**	r/05.05.2020/US/N/10	**3**	3,00E+02				
**156**	w/05.05.2020/US/N/50	**7**	7,00E+02				
**157**	r/02.06.2020/T/NN/10	**1**	1,00E+02	1,00E+02	1,00E+02	1,00E+02	**JUNE (26)**
**158**	r/02.06.2020/T/NN/10	**1**	1,00E+02				
**159**	r/02.06.2020/T/NN/10	**1**	1,00E+02				
**160**	r/02.06.2020/T/NN/50	**1**	1,00E+02				
**161**	r/02.06.2020/K/NN/10	**2**	2,00E+02	1,40E+02	1,00E+02	1,20E+02	
**162**	r/02.06.2020/K/NN/10	**1**	1,00E+02				
**163**	r/02.06.2020/K/NN/10	**1**	1,00E+02				
**164**	w/02.06.2020/K/NN/10	**2**	2,00E+02				
**165**	w/02.06.2020/K/NN/10	**1**	1,00E+02				
**166**	w/02.06.2020/K/NN/50	**1**	1,00E+02				
**167**	w/02.06.2020/K/NN/50	**1**	1,00E+02				
**168**	r/02.06.2020/S/NN/10	**2**	2,00E+02	1,33E+02	1,00E+02	1,17E+02	
**169**	r/02.06.2020/S/NN/10	**1**	1,00E+02				
**170**	r/02.06.2020/S/NN/10	**1**	1,00E+02				
**171**	w/02.06.2020/S/NN/10	**2**	2,00E+02				
**172**	w/02.06.2020/S/NN/10	**1**	1,00E+02				
**173**	r/02.06.2020/S/N/10	**1**	1,00E+02				
**174**	r/02.06.2020/S/N/50	**1**	1,00E+02				
**175**	r/02.06.2020/U/NN/10	**1**	1,00E+02	1,43E+02	1,00E+02	1,21E+02	
**176**	r/02.06.2020/UM/N/10	**2**	2,00E+02				
**177**	w/02.06.2020/UM/N/10	**2**	2,00E+02				
**178**	w/02.06.2020/UM/N/10	**2**	2,00E+02				
**179**	w/02.06.2020/UM/N/10	**1**	1,00E+02				
**180**	w/02.06.2020/UO/N/10	**1**	1,00E+02				
**181**	w/02.06.2020/UO/N/10	**1**	1,00E+02				
**182**	r/02.06.2020/US/N/50	**1**	1,00E+02				
**183**	r/02.07.2020/T/NN/10	**3**	3,00E+02	2,33E+02	6,33E+02	4,33E+02	**JULY (13)**
**184**	r/02.07.2020/T/NN/10	**2**	2,00E+02				
**185**	r/02.07.2020/T/NN/50	**16**	1,60E+03				
**186**	r/02.07.2020/T/NN/50	**2**	2,00E+02				
**187**	w/02.07.2020/T/NN/10	**2**	2,00E+02				
**188**	w/02.07.2020/T/NN/50	**1**	1,00E+02				
**189**	r/02.07.2020/K/NN/50	**3**	3,00E+02	1,33E+02	2,00E+02	1,67E+02	
**190**	w/02.07.2020/K/NN/10	**2**	2,00E+02				
**191**	w/02.07.2020/K/NN/10	**1**	1,00E+02				
**192**	w/02.07.2020/K/NN/10	**1**	1,00E+02				
**193**	r/02.07.2020/S/N/50	**1**	1,00E+02				

**194**	w/02.07.2020/U/NN/10	**1**	1,00E+02	1,00E+02	0,00E+00	5,00E+01	
**195**	r/02.07.2020/UO/N/10	**1**	1,00E+02				
**196**	r/01.08.2020/T/NN/50	**1**	1,00E+02	0,00E+00	1,60E+03	8,00E+02	**AUGUST(34)**
**197**	r/01.08.2020/T/NN/50	**43**	4,30E+03				
**198**	w/01.08.2020/T/NN/50	**4**	4,00E+02				
**199**	r/01.08.2020/K/NN/10	**1**	1,00E+02	2,53E+03	6,00E+02	1,57E+03	
**200**	r/01.08.2020/K/NN/10	**69**	6,90E+03				
**201**	r/01.08.2020/K/NN/50	**10**	1,00E+03				
**202**	w/01.08.2020/K/NN/10	**6**	6,00E+02				
**203**	w/01.08.2020/K/NN/50	**2**	2,00E+02				
**204**	r/01.08.2020/S/NN/10	**1**	1,00E+02	9,33E+02	2,92E+03	1,93E+03	
**205**	r/01.08.2020/S/NN/10	**1**	1,00E+02				
**206**	r/01.08.2020/S/NN/10	**1**	1,00E+02				
**207**	w/01.08.2020/S/NN/10	**41**	4,10E+03				
**208**	w/01.08.2020/S/NN/10	**11**	1,10E+03				
**209**	w/01.08.2020/S/NN/10	**1**	1,00E+02				
**210**	r/01.08.2020/S/N/50	**12**	1,20E+03				
**211**	w/01.08.2020/S/N/50	**16**	1,60E+03				
**212**	w/01.08.2020/S/N/50	**63**	6,30E+03				
**213**	w/01.08.2020/S/N/50	**3**	3,00E+02				
**214**	w/01.08.2020/S/N/50	**52**	5,20E+03				
**215**	r/01.08.2020/U/NN/10	**1**	1,00E+02	2,50E+02	6,71E+02	2,55E+02	
**216**	r/01.08.2020/U/NN/10	**1**	1,00E+02				
**217**	r/01.08.2020/U/NN/10	**2**	2,00E+02				
**218**	r/01.08.2020/UM/N/10	**1**	1,00E+02				
**219**	r/01.08.2020/UM/N/50	**1**	1,00E+02				
**220**	w/01.08.2020/UM/N/10	**1**	1,00E+02				
**221**	w/01.08.2020/UM/N/50	**33**	3,30E+03				
**222**	w/01.08.2020/UM/N/50	**7**	7,00E+02				
**223**	w/01.08.2020/UM/N/50	**3**	3,00E+02				
**224**	w/01.08.2020/UM/N/50	**1**	1,00E+02				
**225**	w/01.08.2020/UM/N/50	**1**	1,00E+02				
**226**	r/01.08.2020/UO/N/50	**1**	1,00E+02				
**227**	w/01.08.2020/UO/N/10	**5**	5,00E+02				
**228**	w/01.08.2020/UO/N/10	**8**	8,00E+02				
**229**	r/01.08.2020/US/N/10	**1**	1,00E+02				
**230**	w/06.09.2020/T/NN/10	**1**	1,00E+02	1,00E+02	0,00E+00	5,00E+01	**SEPTEMBER (20)**
**231**	w/06.09.2020/K/NN/10	**1**	1,00E+02	1,00E+02	0,00E+00	5,00E+01	
**232**	r/06.09.2020/S/NN/10	**300**	3,00E+04	4,41E+03	1,00E+02	2,26E+03	
**233**	r/06.09.2020/S/NN/10	**2**	2,00E+02				
**234**	r/06.09.2020/S/NN/10	**1**	1,00E+02				
**235**	r/06.09.2020/S/NN/50	**1**	1,00E+02				
**236**	w/06.09.2020/S/NN/10	**1**	1,00E+02				
**237**	w/06.09.2020/S/NN/10	**1**	1,00E+02				
**238**	w/06.09.2020/S/NN/50	**1**	1,00E+02				
**239**	w/06.09.2020/S/N/10	**1**	1,00E+02				
**240**	w/06.09.2020/S/N/10	**3**	3,00E+02				
**241**	r/06.09.2020/U/NN/50	**7**	7,00E+02	4,40E+02	1,30E+03	8,70E+02	
**242**	w/06.09.2020/U/NN/50	**1**	1,00E+02				
**243**	r/06.09.2020/UM/N/10	**18**	1,80E+03				
**244**	w/06.09.2020/UM/N/10	**1**	1,00E+02				
**245**	r/06.09.2020/UO/N/50	**17**	1,70E+03				
**246**	w/06.09.2020/UO/N/10	**1**	1,00E+02				

**247**	w/06.09.2020/UO/N/10	**1**	1,00E+02				
**248**	r/06.09.2020/US/N/10	**1**	1,00E+02				
**249**	r/06.09.2020/US/N/50	**27**	2,70E+03				
**250**	r/01.10.2020/S/NN/10	**2**	2,00E+02	1,44E+02	1,00E+02	1,22E+02	**OCTOBER (10)**
**251**	r/01.10.2020/S/NN/10	**1**	1,00E+02				
**252**	r/01.10.2020/S/NN/10	**3**	3,00E+02				
**253**	w/01.10.2020/S/NN/10	**2**	2,00E+02				
**254**	r/01.10.2020/UO/N/10	**1**	1,00E+02				
**255**	r/01.10.2020/UO/N/50	**1**	1,00E+02				
**256**	w/01.10.2020/UO/N/10	**1**	1,00E+02				
**257**	w/01.10.2020/UO/N/10	**1**	1,00E+02				
**258**	w/01.10.2020/UO/N/10	**1**	1,00E+02				
**259**	w/01.10.2020/US/N/10	**1**	1,00E+02				

Sampling code structure: time of day / date / lake / supervision type / sampling depth (cm); where:

– r = morning, w = evening;

– date = day.month.year (e.g., 04.04.19);

– lake = lake adjacent to the sampled beach (e.g., T = Tyrsko, S = Skanda, etc.);

– supervision type: N = supervised beach, NN = unsupervised beach;

– sampling depth = depth of sand sampling in centimeters (e.g., 10 or 50).
